# Health systems and quality of life: The situation of South Africans with spinal cord injury

**DOI:** 10.4102/sajp.v81i1.2133

**Published:** 2025-06-06

**Authors:** Eugene Nizeyimana, Anthea Rhoda, Joyce Mothabeng, Francois Theron, Conran Joseph

**Affiliations:** 1 Department of Health and Rehabilitation Sciences, Faculty of Medicine and Health Science, Stellenbosch University, Cape Town, South Africa; 2 Department of Physiotherapy, Faculty of Community and Health Sciences, University of the Western Cape, Cape Town, South Africa; 3 Department of Physiotherapy, Faculty of Health Sciences, University of Pretoria, Pretoria, South Africa; 4 Meulmed Rehabilitation Hospital, Pretoria, South Africa; 5 Department of Physiotherapy, Faculty of Medicine and Health Sciences, Stellenbosch University, Cape Town, South Africa

**Keywords:** health systems, quality of life, social factors, spinal cord injury, South Africa

## Abstract

**Background:**

Spinal cord injury (SCI) significantly impacts quality of life (QoL) through physical disabilities and reduced social participation.

**Objectives:**

This study compared health system indicators, social factors and self-reported QoL between individuals with SCI accessing public versus private healthcare in South Africa.

**Method:**

A cross-sectional survey using the International Spinal Cord Injury (InSCI) community survey questionnaire was conducted with 200 SCI individuals (156 public, 44 private sector) from Cape Town and Pretoria. Chi-square tests and correlation analyses were performed.

**Results:**

Significant disparities were observed between cohorts. Public sector participants reported higher rates of disability pension receipt (82.1% vs 54.5%) and greater challenges accessing various services. Overall, self-reported QoL was 56%, with the private cohort reporting significantly higher satisfaction (64% vs 52%). Private sector participants also reported higher satisfaction with living conditions and personal relationships. Access to disability pension and healthcare negatively correlated with QoL, while access to public spaces, medication, transport and nursing care positively correlated with QoL.

**Conclusion:**

This study reveals significant disparities in health system performance, social factors and QoL between SCI individuals accessing public versus private healthcare in South Africa.

**Clinical implications:**

Findings highlight the need to address systemic inequities in healthcare access and social support for individuals with SCI to improve QoL across sectors.

## Introduction

Spinal cord injury (SCI) represents a catastrophic event that precipitates permanent physical disabilities with profound implications for patients’ functional capabilities and psychological well-being (Charlifue et al. 2012). The resultant impairments significantly attenuate social participation and economic productivity, generating a substantial societal burden (Wang et al. 2025). These multidimensional challenges frequently precipitate social isolation and psychological distress, including depression and anxiety, further compromising overall well-being and quality of life (QoL) (Fehlings et al. 2017).

The concept of QoL encompasses a complex, multifaceted phenomenon reflecting the holistic well-being of individuals within societal contexts (Petrovič & Maturkanic, 2022). According to the World Health Organization (WHO), QoL represents an individual’s subjective assessment of their position in life relative to their cultural framework and value systems, particularly concerning personal goals, expectations, standards and preoccupations (Bujang et al. 2023; WHO 1988).

Within this broader conceptual landscape, health-related quality of life (HRQoL) emerges as a more specific construct focused on the optimisation of mental, physical and role-functioning domains. Health-related quality of life encompasses occupational performance while simultaneously addressing interpersonal relationship quality and subjective perceptions of health status, such as physical fitness, life satisfaction and general well-being (Theofilou 2013). The relationship between these constructs warrants careful delineation. Health-related quality of life specifically integrates an individual’s satisfaction with treatment outcomes, current health status and future health prospects, creating a health-centric evaluation framework. In contrast, the broader QoL concept transcends health parameters to incorporate additional dimensions, including housing adequacy, income sufficiency and environmental perceptions (Theofilou 2013).

This conceptual hierarchy positions life satisfaction as a cognitive-evaluative component within the broader QoL framework, while HRQoL represents a domain-specific application focusing on health-related dimensions that influence the overall quality of life. For individuals with SCI, these constructs interact dynamically. Physical limitations impact functional capacity HRQoL, which subsequently influences global life satisfaction and ultimately shapes overall QoL. Life satisfaction, as a cognitive component within this framework, constitutes a global assessment of one’s overall QoL (Malvaso & Kang 2022).

These theoretical frameworks underscore the imperative for developing healthcare and social service interventions that strategically target comprehensive QoL enhancement for individuals with SCI. This patient-centred approach acknowledges that meaningful clinical outcomes must transcend physiological parameters to address the holistic impact of interventions on functional capacity and subjective well-being across multiple life domains.

For individuals with SCI in South Africa, particularly those relying on public healthcare, QoL is compromised by inadequate health system performance, including limited access to comprehensive care along the injury trajectory. Challenges in obtaining specialised nursing care, essential medications, medical supplies and assistive technologies further exacerbate these difficulties (Hanass-Hancock et al. 2017; Moller & Taule 2012; Vergunst et al. 2015). Contextual barriers such as inaccessible public spaces, inadequate transportation and negative societal attitudes also complicate post-SCI adjustment and reintegration (Nizeyimana et al. 2024). These combined factors contribute to reduced independence, social isolation, poor health outcomes and decreased QoL (Bezuidenhout et al. 2023).

In public health, QoL is a vital indicator of service needs and utilisation for individuals with disabilities, including those with SCI (Borg et al. 2020). While healthcare system performance significantly impacts QoL, it is also shaped by social determinants, genetic predispositions and environmental factors (Fink, Keyes & Cerdá 2016). Effective intersectoral coordination across health, social welfare and labour sectors, along with equitable service access, is essential for promoting recovery, social reintegration and sustainable livelihoods.

In South Africa, healthcare is divided into public and private sectors. Public healthcare, which is government funded and theoretically accessible to all citizens, faces challenges such as resource constraints, medication shortages and limited personnel, impacting system performance (Young 2016). In contrast, private healthcare offers specialised care, multidisciplinary teams, advanced equipment and shorter waiting times but remains costly and dependent on medical-aid coverage (Young 2016). Many patients with limited insurance initially access private care but must transition to public facilities when funds are depleted, disrupting the continuity of care. Thus, the healthcare system faces challenges, including unequal resource distribution, poor management, leadership crises and slow restructuring (Naidoo 2012), exacerbating disparities and limiting access to quality services.

Previous research has linked access to private health insurance with higher satisfaction regarding services and facilities among persons with disabilities in South Africa (Statistics South Africa 2014). This suggests that health systems significantly impact service satisfaction. However, what remains unknown is how individuals with SCI specifically experience services and facilities across these two healthcare sectors and the extent to which these experiences influence their levels of life satisfaction and QoL. To address these knowledge gaps, this study aimed to: (1) determine the extent to which selected healthcare indicators have been met and the influence of social factors as perceived by persons with SCI; (2) assess self-reported QoL in a sample of persons with SCI; (3) evaluate differences in selected healthcare indicators, social factors influence and self-reported QoL between persons with SCI accessing public versus private healthcare and (4) examine the relationship between selected health system indicators, social factors and self-reported QoL. Understanding the interplay between selected healthcare systems, social factors and QoL is crucial for developing targeted interventions and policies aimed at improving the lives of individuals with SCI.

## Research methods and design

### Setting

Data were collected within communities in the metropolitan areas of Cape Town and Pretoria in the South African provinces of the Western Cape and Gauteng, respectively. The two provincial geographical settings differ in terms of healthcare system governance, availability and access to services. SCI participants utilising public healthcare services in Cape Town, and those accessing private healthcare in Pretoria were conveniently recruited, reflecting South Africa’s parallel healthcare systems and addressing knowledge gaps in spinal cord injury research. While the uneven distribution (156 public vs 44 private participants) affects precision, it aligns with the established 3:1 public-private incidence ratio documented by Phillips, Braaf and Joseph (2018). This approach, though limiting extrapolation to other facilities, provides valuable insights into both healthcare systems despite methodological constraints.

### Study design and sample

This study employed a descriptive cross-sectional survey for data collection. Participants were selected using non-probabilistic sampling and included individuals aged 18 years or older with a confirmed primary diagnosis of either traumatic or non-traumatic SCI, at least 1-year post-injury. Recruitment was conducted through public and private healthcare facility registries. Individuals with severe cognitive impairment or those hospitalised during the survey period were excluded from the study.

The Cape Town cohort (*N* = 156) consisted of individuals accessing public healthcare and was originally part of a prior epidemiological study (Joseph et al. 2015). Following ethical approval for the current study, all previous participants were invited to take part in a cross-sectional study examining functioning, healthcare systems and social determinants influencing QoL in individuals with SCI. Of the 145 participants from the earlier study, 87 were available for follow-up. An additional 69 new participants were recruited from outpatient services at the Western Cape Rehabilitation Centre and through regional peer support networks.

The private healthcare cohort (*N* = 44) comprised individuals who underwent inpatient rehabilitation at Mediclinic Muelmed but were discharged at the time of the study. Because the hospital maintained a patient registry and data collectors were unfamiliar with Gauteng province, hospital administrators arranged for interviews to be conducted in a secure and private room within the facility to ensure the safety of both researchers and participants.

### Procedure and instruments

Two participants chose to complete the survey at the rehabilitation facility, while the rest preferred telephone interviews. Data collection was conducted using the International Spinal Cord Injury (InSCI) community survey questionnaire, which is based on two fundamental frameworks: the International Classification of Functioning, Disability and Health (ICF) and the Health Core Sets for Spinal Cord Injury (SCI). The survey’s development process has been extensively documented in previous research (Fekete et al. 2017; Gross-Hemmi et al. 2017).

The InSCI questionnaire consists of 125 items structured into six key components, capturing the lived experiences of individuals with SCI. These components include but are not limited to functioning (body functions and structures) with 28 questions, activities and participation with 42 questions and contextual factors, which are divided into environmental factors (26 questions) and personal factors (19 questions). Additionally, self-reported QoL is assessed through five questions. This comprehensive framework enables a holistic evaluation of the challenges and experiences of individuals living with SCI.

### Quality of life and health system indicators

Data on QoL and satisfaction were collected using the World Health Organization QoL BREF (WHO-QoL-BREF) section within the InSCI questionnaire. The WHO-QoL-BREF assesses self-reported QoL through five items, measuring satisfaction with health, daily activities, self-perception, personal relationships and living conditions. This validated tool has been widely used across several contexts to facilitate population comparisons (Biering-Sørensen et al. 2011). Participants rated their QoL over the previous 14 days using a 5-point Likert scale (1 = very dissatisfied to 5 = very satisfied), and an overall QoL score was derived from these responses (Fekete et al. 2017).

Health system indicators and social factors, selected from the International Spinal Cord Injury Survey, were linked to WHO-QoL-BREF items based on recommendations from the International Perspectives of Spinal Cord Injury (Biering-Sørensen et al. 2011; Sullivan & Artino 2013). These indicators included:

- Receipt of disability pension or similar benefits because of inability to work.
- Participation in vocational rehabilitation post-SCI.
- Unmet healthcare needs in the past 12 months.
- Accessibility of public spaces (e.g., buildings and parks).
- Social attitudes, including stigma and prejudice.
- Availability of assistive devices (e.g., wheelchairs and walking aids).
- Access to transportation (e.g., adapted vehicles and public transit usability).
- Reception of nursing care (e.g., home healthcare and personal assistance).
- Availability of medication, aids and medical supplies (e.g., catheters and splints).

Responses to the first three items were binary (yes/no), while items 4–9 assessed whether the absence or inadequacy of these resources negatively impacted participants’ lives or had no effect. The survey was available in English, Afrikaans and isiXhosa and required approximately 45–60 min to complete.

### Data analysis

The analyses were conducted using the Statistical Package for Social Sciences (SPSS) Version 28. Descriptive statistics was used to summarise demographic variables (age, sex, marital status, educational level, employment status and household income) and injury-related characteristics (type, level, severity of injury and duration since injury). Results were reported as frequencies and percentages.

Responses for health system indicators and social factors were dichotomised, with positive outcomes (e.g., no impact, access granted and services received) coded as 1 and negative outcomes (e.g., impact present, no access and services not received) coded as 0. Similarly, self-reported QoL responses from the 5-point Likert scale (1 = very dissatisfied to 5 = very satisfied) were categorised as either positive (satisfied or very satisfied, coded as 1) or negative (neutral, dissatisfied or very dissatisfied, coded as 0). An overall QoL score was calculated using the WHO methodology (WHO-WHOQOL), generating a total score ranging from 0% to 100%, where higher values indicate better QoL.

Frequencies and percentages of dichotomised responses for health system indicators, social factors and self-reported QoL were computed. Comparisons between the public and private healthcare cohorts were conducted using cross-tabulation and the Chi-square test of independence. Additionally, correlation analyses were performed to examine relationships between health system indicators, social factors and self-reported QoL within the study sample. A *p*-value of ≤ 0.05 was considered statistically significant.

### Ethical considerations

The ethics committee of the University of the Western Cape approved this study (BM/16/3/24). The written consent to participate was obtained from the respondents who participated in the study.

## Results

### Socio-demographic characteristics

The study included 200 SCI participants from both public (*n* = 156) and private (*n* = 44) healthcare facilities. The majority (78%) were from the public cohort, and 75% were male. The median age was 36 years, and the median duration of the injury was 6 years. Traumatic injuries were the primary cause (92%) across both cohorts, with paraplegia and complete injuries being the most common diagnoses. Only 9% of participants had a university education. The private cohort had a higher percentage of employed participants (57%). Most reported a monthly household income between R1000.00 and R3000.00. However, a significant income disparity was found as 64% of private cohort participants earned over R20 000.00 compared to just 4% in the public cohort (*p* = 0.000). Table 1 provides further demographic and injury details.

**TABLE 1:** Demographics and injury characteristics of the study sample.

Variables	Public *N* = 156				Private *N* = 44				All *N* = 200				*p*
	Mean	Median	*n*	%	Mean	Median	*n*	%	Mean	Median	*n*	%	
Age at time of survey	35	43	-	-	32	42	-	-	37	36	-	-	0.000
**Gender**	-	-	-	-	-	-	-	-	-	-	-	-	0.430
Male	-	-	119	76.3	-	-	31	70.5	-	-	150	25.0	-
Female	-	-	37	23.7	-	-	13	29.5	-	-	50	25.0	-
**Age category (years)**	-	-	-	-	-	-	-	-	-	-	-	-	0.014
18–30	-	-	68	43.5	-	-	7	15.9	-	-	75	37.0	-
31–45	-	-	50	32.7	-	-	18	40.9	-	-	68	34.5	-
46–60	-	-	30	19.2	-	-	15	34.1	-	-	45	22.5	-
61–75	-	-	8	5.1	-	-	4	9.1	-	-	12	6.0	-
**Marital status**	-	-	-	-	-	-	-	-	-	-	-	-	**0.000**
Single	-	-	116	74.4	-	-	19	43.1	-	-	135	67.5	-
Married	-	-	17	10.9	-	-	16	36.4	-	-	33	16.5	-
Separated	-	-	23	14.7	-	-	9	20.5	-	-	32	16.0	-
Divorced or widowed	-	-			-	-	-	-	-	-	-	-	-
**Aetiology**	-	-	-	-	-	-	-	-	-	-	-	-	0.400
Traumatic	-	-	143	91.7	-	-	42	95.5	-	-	185	92.5	-
Non-traumatic	-	-	13	8.3	-	-	2	4.5	-	-	15	7.5	-
**Injury level**	-	-	-	-	-	-	-	-	-	-	-	-	0.179
Paraplegia	-	-	99	63.5	-	-	23	52.3	-	-	122	61.0	-
Tetraplegia	-	-	57	36.5	-	-	21	47.7	-	-	78	39.0	-
**Completeness**	-	-	-	-	-	-	-	-	-	-	-	-	0.253
Complete	-	-	79	50.6	-	-	26	60.5	-	-	105	52.8	-
Incomplete	-	-	77	49.4	-	-	17	39.5	-	-	95	47.2	-
**Chronicity (years)**	-	-	-	-	-	-	-	-	-	-	-	-	**0.028**
< 1	-	-	11	7.1	-	-	6	13.6	-	-	17	8.5	-
1–2	-	-	17	10.9	-	-	9	20.5	-	-	26	13.0	-
3–5	-	-	46	9.5	-	-	6	13.6	-	-	52	26.0	-
6–10	-	-	31	19.9	-	-	4	9.1	-	-	35	17.5	-
>10	-	-	51	32.7	-	-	19	43.2	-	-	70	35.0	-
**Chronicity**	8	5	-	-	10	9	-	-	9	6	-	-	0.860
**Main causes**	-	-	-	-	-	-	-	-	-	-	-	-	**0.004**
Assault	-	-	71	45.5	-	-	10	22.7	-	-	81	40.5	-
Traffic	-	-	54	34.6	-	-	16	36.3	-	-	70	35.0	-
Sports	-	-	24	15.4	-	-	10	22.7	-	-	34	17.0	-
Infection	-	-	7	4.5	-	-	8	18.3	-	-	15	7.5	-
**Education**	-	-	-	-	-	-	-	-	-	-	-	-	**0.018**
Primary	-	-	16	10.5	-	-	1	2.2	-	-	17	8.5	-
Secondary (low/high)	-	-	118	75.6	-	-	18	40.9	-	-	136	68.0	-
Post-secondary	-	-	18	11.5	-	-	10	22.9	-	-	28	14.0	-
Tertiary	-	-	0	0.0	-	-	19	36.4	-	-	19	9.5	-
**Employment**	-	-	-	-	-	-	-	-	-	-	-	-	**0.000**
Worked before injury	-	-	83	53.5	-	-	37	84.1	-	-	120	60.3	-
Paid work currently	-	-	24	15.4	-	-	25	56.8	-	-	49	24.5	-
**Household income**	-	-	-	-	-	-	-	-	-	-	-	-	**0.000**
R1000.00–R3000.00	-	-	79	50.7	-	-	7	16.0	-	-	86	43.0	-
R3001.00–R9000.00	-	-	53	34.0	-	-	2	4.0	-	-	55	27.5	-
R9001.00–R20 000.00	-	-	17	10.9	-	-	7	16.0	-	-	24	12.0	-
R20 001.00–R50 000.00	-	-	7	4.4	-	-	15	34.0	-	-	22	11.0	-
> R50 000.00	-	-	0	0.0	-	-	13	30.0	-	-	13	6.5	-

Note: The bold values indicate the statistically significant values at *p* < 0.05.

### Health system indicators and social factors

A notable discrepancy in disability pension receipt was observed, with 82.1% of public sector respondents receiving it compared to 54.5% in the private sector. Vocational rehabilitation services were utilised by 60% of public sector participants, compared to 43.2% in the private sector, with a significant *p*-value of 0.042. Issues such as lack of transport, medications, assistive devices and nursing care were reported more frequently by the public sector participants than those in the private sector. No significant differences were found between the two groups for the indicators ‘needed healthcare’, ‘inaccessibility to public spaces’ and ‘negative societal attitudes’. Table 2 illustrates the differences in health system indicators and social factors between public and private healthcare users.

**TABLE 2:** Health system indicator and social factor scores.

Variables	Public		Private		All value		*p*
	*n*	%	*n*	%	*n*	%	
**Disability pension**	-	-	-	-	-	-	**0.000**
Currently receiving	128	82.1	24	54.5	152	76.0	-
Currently not receiving	28	17.9	20	45.5	48	24.0	-
**Vocational rehabilitation**	-	-	-	-	-	-	**0.042**
Received after SCI	93	60.0	19	43.2	112	57.0	-
Did not receive after SCI	63	40.0	25	54.8	88	43.0	-
**Needed healthcare**	-	-	-	-	-	-	0.396
Received it	111	71.2	34	77.3	145	72.5	-
Did not receive it	45	28.8	10	22.7	55	27.5	-
**Inaccessible public space**	-	-	-	-	-	-	0.459
Made life harder	110	71.4	29	65.9	139	70.2	-
Had no influence	44	28.6	15	43.1	59	29.8	-
**Lack of transport**	-	-	-	-	-	-	**0.002**
Made life harder	98	62.6	16	36.4	114	56.8	-
Had no influence	58	37.4	28	63.6	86	43.2	-
**Lack of medications**	-	-	-	-	-	-	**0.008**
Made life harder	96	61.3	17	38.6	113	56.3	-
Had no influence	60	38.7	27	61.4	87	43.7	-
**Insufficient assistive devices**	-	-	-	-	-	-	**0.003**
Made life harder	93	59.4	15	34.1	108	53.8	-
Had no influence	63	40.6	29	65.9	92	46.2	-
**Insufficient nursing care**	-	-	-	-	-	-	**0.000**
Made life harder	83	52.9	8	18.2	91	45.2	-
Had no influence	73	47.1	36	81.8	109	54.8	-
**Negative societal attitudes**	-	-	-	-	-	-	0.263
Made life harder	69	43.5	15	34.1	83	41.4	-
Had no influence	87	56.5	29	65.9	116	58.6	-

Note: The bold values indicate the statistically signicant values at *p* < 0.05.

SCI, spinal cord injury.

### Self-reported quality of life

Table 3 presents the self-reported QoL results, highlighting differences between individuals with public and private healthcare insurance. Overall, 56% of participants expressed satisfaction, with the private cohort reporting significantly higher satisfaction (64%) compared to the public cohort (52%). Satisfaction with oneself (72%) and satisfaction with health (69%) were the highest-rated areas, with the private cohort reporting higher scores, though these differences were not statistically significant (*p* = 0.366 and 529, respectively). The lowest-rated aspect was satisfaction with living conditions (59%), with the public cohort giving the lowest score. Generally, participants in the private cohort reported higher QoL scores. Significant differences favouring the private cohort were found in overall satisfaction, satisfaction with living conditions and satisfaction with personal relationships.

**TABLE 3:** Self-reported quality of life.

Variables	Public		Private		All		*p*
	*n*	%	*n*	%	*n*	%	
**Satisfaction with health**	-	-	-	-	-	-	0.529
Satisfied	106	67.7	32	72.7	138	68.8	-
Dissatisfied	50	32.3	12	31.2	62	31.2	-
**Satisfaction with daily routine**	-	-	-	-	-	-	0.106
Satisfied	101	64.3	34	77.3	135	67.2	-
Dissatisfied	55	35.7	10	22.7	65	32.8	-
**Satisfaction with oneself**	-	-	-	-	-	-	0.366
Satisfied	109	70.3	34	77.3	143	71.9	-
Dissatisfied	47	29.7	10	22.7	57	28.1	-
**Satisfaction with relationships**	-	-	-	-	-	-	**0.004**
Satisfied	96	61.0	37	84.0	133	66.5	-
Dissatisfied	60	39.0	7	16.0	67	33.5	-
**Satisfaction with living conditions**	-	-	-	-	-	-	**0.000**
Satisfied	82	52.6	38	84.0	120	59.6	-
Dissatisfied	74	47.4	6	16.0	80	40.4	-
**Overall satisfaction (0 % – 100 %)**	**52**	-	**64**	-	**56**	-	-

Note: The bold values indicate the statistically signicant values at *p* < 0.05.

### Relationship between health system indicators, social factors and self-reported quality of life

The relationship between health system indicators, social factors and self-reported QoL was examined, and the results are presented in Table 4. Access to disability pension and healthcare were negatively correlated with self-reported QoL. There was a positive correlation between self-reported QoL and access to public space, medication, transport and nursing care.

**TABLE 4:** Relationship between health system indicators, social factors and self-reported quality of life of the study sample.

Health systems and social factors	Self-reported quality of life	*p*
Access to vocational rehabilitation	0.075	0.293
Access to disability pension	−0.235	**0.001**
Access to healthcare	−0.150	**0.034**
Access to public space	0.145	**0.042**
Access to medication	0.186	**0.008**
Access to assistive devices	0.128	0.072
Access to transport	0.228	**0.001**
Access to nursing care	0.195	**0.006**
Societal attitude	0.115	0.108

Note: The bold values indicate the statistically signicant values at *p* < 0.05; Reported are correlation coefficient.

Overall, the study revealed significant disparities between public and private SCI healthcare users, with the private cohort experiencing higher income, employment rates and QoL. Despite no differences in access to some services, socioeconomic factors varied markedly between the two groups.

## Discussion

This study is the first to compare health system indicators, social factors and self-reported QoL for individuals with SCI using public or private healthcare in South Africa. Significant demographic differences were found, with private sector patients being older, more likely to be married and have higher employment rates, which could impact rehabilitation outcomes independent of the healthcare setting. Additionally, the higher incidence of assault-related injuries in the public sector, compared to traffic accidents and infections in the private sector, adds complexity. Assault cases often stem from challenging social conditions and violence, which can affect QoL. Future analyses should control these demographic and aetiological factors to more accurately interpret sectoral differences in rehabilitation outcomes.

The study highlights significant disparities in health system indicators, social factors influence and QoL between the two cohorts. Public sector participants, who are more dependent on disability pensions and face greater challenges accessing essential services, such as nursing care and assistive devices, report lower overall satisfaction and QoL. In contrast, private sector participants exhibit higher satisfaction with living conditions, personal relationships and overall QoL. These findings underscore the importance of addressing systemic inequities in healthcare access and social support to improve outcomes for individuals with SCI.

The study found an overall self-reported QoL of 56%, with the private cohort reporting significantly higher QoL than the public cohort, aligning with a 2014 Statistics South Africa report showing 91% satisfaction in private healthcare users compared to 54% in public healthcare users. This disparity may be because of financial status, as 64% of the private cohort had household incomes of R20 000.00 or more, compared to only 4% in the public cohort. Previous research (Shim & Park 2020; Singh & Cleveland 2020) has shown that higher household incomes correlate with better QoL for individuals with SCI. Thus, the higher incomes noted in the private cohort likely enabled access to higher-quality private healthcare leading to better satisfaction and QoL. These findings indicate that socioeconomic factors, like income and healthcare access, significantly affect SCI outcomes in South Africa. To improve QoL for all individuals, efforts must focus on financial empowerment and improving subsidised medications, accessible facilities, assistive devices, community rehabilitation and education. This dual approach recognises that economic empowerment must be complemented by systemic healthcare improvements to enhance QoL for all individuals with SCI regardless of socioeconomic status.

This study found a higher proportion of disability pension recipients in the public cohort (76%) than in the private cohort, likely because of lower employment rates in the public sector (15% vs 57%). Hanass-Hancock et al. (2017) highlighted the reliance on disability grants for individuals with SCI because of high unemployment. Notably, our study revealed a negative correlation between receiving a disability pension and self-reported QoL, as public cohort participants, who mainly received disability pensions, reported lower satisfaction than the private cohort. In contrast, private cohort participants reported higher incomes, less reliance on disability pensions and better QoL. These findings align with Fekete et al. (2014), who found a link between household income and life satisfaction among individuals with disabilities in Switzerland. This suggests that household income may have a stronger positive impact on QoL than disability pensions, particularly in South Africa, where financial challenges are greater, and the disability pension (less than R3000.00) is insufficient for SCI-related expenses.

Accessible public spaces and transportation are essential for social participation, community engagement and independent living. However, participants in the public cohort reported significant barriers related to inaccessible spaces, inadequate transport and limited healthcare access, which affected their daily lives. These findings are consistent with previous studies (Bezuidenhout et al. 2023; Nizeyimana et al. 2024; Vanderschuren & Nnene 2021; Vergunst et al. 2017; Visagie et al. 2017), which highlighted how environmental obstacles, such as inaccessible buildings and transport, hinder the participation of individuals with SCI in various life activities. These barriers can lead to social isolation, reduced healthcare access, limited employment opportunities and a lower QoL. The persistence of these issues emphasises the need for comprehensive strategies to enhance accessibility and inclusion in South African communities.

### Study limitations

Although this study demonstrates significant differences between public and private cohorts regarding health system indicators, social factors and self-reported QoL, a considerable discrepancy in group sample sizes occurred. Thus, the capacity of the study to determine the relationship between health system indicators, social factors and QoL while moderating the effect of access to healthcare insurance was limited. Therefore, comparing the results based on the private and public cohorts should be interpreted with discretion.

The study employed convenience sampling across two provinces and healthcare systems, limiting the generalisability of findings. While this allowed valuable comparison between public and private sectors, results may not represent the broader South African spinal cord injury population. Future research should employ more rigorous sampling strategies across additional provinces.

Furthermore, the absence of a national population-based registry for SCI in South Africa directly challenges the generalisability of the study findings. However, because no significant differences exist, in terms of demographic and injury-related factors, when compared our study to previous reports from local settings (Joseph et al. 2015), this study may be generalisable to the source population.

## Conclusion

This study explores the complex landscape of healthcare experiences and QoL among persons with SCI in South Africa, highlighting the multifaceted nature of healthcare system performance and social factors. While the research revealed differences between public and private healthcare sectors, careful interpretation is crucial given the study’s methodological limitations.

The observed variations in health system indicators and self-reported QoL between public and private sector participants should not be simplistically attributed solely to healthcare sector differences. Notably, the significant discrepancy in sample sizes and the lack of pre-injury baseline data warrant caution against drawing definitive conclusions about the comprehensive quality of healthcare services. The findings underscore the importance of recognising the intricate interplay between healthcare access, social support and individual experiences.

The study suggests that socioeconomic factors and pre-existing conditions likely contribute substantially to the observed differences in QoL and social factors. These disparities may have existed before participants’ interactions with healthcare systems, indicating a broader societal context of inequality that extends beyond healthcare provision. The research points to the need for a more holistic approach to understanding the challenges faced by individuals with SCI.

The findings highlight the need for comprehensive, context-sensitive interventions to address systemic inequities. Policymakers and healthcare providers should consider the broader social determinants of health, recognising that multiple interconnected factors beyond the healthcare sector alone influence QoL.

While the current study provides valuable insights, it also underscores the need for more sophisticated, context-aware research methodologies. The goal should be to develop targeted, equitable interventions that support all individuals with SCI, regardless of their healthcare sector or socioeconomic background. Addressing the complex challenges faced by this vulnerable population requires a multifaceted, empathetic approach that goes beyond simplistic sector-based comparisons. Future research should prioritise longitudinal studies with comparable sample sizes, comprehensive baseline assessments before the injury and in-depth exploration of pre-existing social and economic factors.

## References

[CIT0001] Bezuidenhout, L., Rhoda, A., Moulaee Conradsson, D., Theron, F. & Joseph, C., 2023, ‘Factors influencing employment among people with spinal cord injury in South Africa’, *Disability and Rehabilitation* 45(26), 4381–4387. 10.1080/09638288.2022.2151651 36447405

[CIT0002] Biering-Sørensen, F., Bickenbach, J.E., El Masry, W.S., Officer, A. & Von Groote, P.M., 2011, ‘ISCoS–WHO collaboration. International perspectives of spinal cord injury (IPSCI) report’, *Spinal Cord* 49(6), 679–683. 10.1038/sc.2011.12 21423254

[CIT0003] Borg, D.N., Foster, M.M., Legg, M., Jones, R., Kendall, E., Fleming, J. et al., 2020, ‘The effect of health service use, unmet need, and service obstacles on quality of life and psychological well-being in the first year after discharge from spinal cord injury rehabilitation’, *Archives of Physical Medicine and Rehabilitation* 101(7), 1162–1169. 10.1016/j.apmr.2020.02.008 32145278

[CIT0004] Bujang, M.A., Lai, W.H., Hon, Y.K., Yap, E.P.P., Tiong, X.T. et al., 2023, ‘Measuring population health and quality of life: Developing and testing of the significant quality of life measure (SigQOLM)’, *Heliyon* 9(12), e22668. 10.1016/j.heliyon.2023.e22668 38149205 PMC10750041

[CIT0005] Charlifue, S., Post, M.W., Biering-Sørensen, F., Catz, A., Dijkers, M., Geyh, S. et al., 2012, ‘International spinal cord injury quality of life basic data set’, *Spinal Cord* 50(9), 672–675. 10.1038/sc.2012.27 22450884

[CIT0006] Fehlings, M.G., Tetreault, L.A., Wilson, J.R., Kwon, B.K., Burns, A.S., Martin, A.R. et al., 2017, ‘A clinical practice guideline for the management of acute spinal cord injury: Introduction, rationale, and scope’, *Global Spine Journal* 7(3_suppl), 84S–94S. 10.1177/2192568217703387 PMC5684846 29164036

[CIT0007] Fekete, C., Post, M.W., Bickenbach, J., Middleton, J., Prodinger, B., Selb, M. et al., 2017, ‘A structured approach to capture the lived experience of spinal cord injury: Data model and questionnaire of the international spinal cord injury community survey’, *American Journal of Physical Medicine & Rehabilitation* 96(2), S5–S16. 10.1177/2192568217703387 28059874

[CIT0008] Fekete, C., Siegrist, J., Reinhardt, J.D., Brinkhof, M.W. & SwiSCI Study Group, 2014, ‘Is financial hardship associated with reduced health in disability? The case of spinal cord injury in Switzerland’, *PLoS One* 9(2), e90130. 10.1371/journal.pone.0090130 24587239 PMC3938582

[CIT0009] Fink, D.S., Keyes, K.M. & Cerdá, M., 2016, ‘Social determinants of population health: A systems sciences approach’, *Current Epidemiology Reports* 3(1), 98–105. 10.1007/s40471-016-0066-8 27642548 PMC5025257

[CIT0010] Gross-Hemmi, M.H., Post, M.W., Ehrmann, C., Fekete, C., Hasnan, N., Middleton, J.W. et al., 2017, ‘Study protocol of the international Spinal Cord Injury (InSCI) community survey’, *American Journal of Physical Medicine & Rehabilitation* 96(2), S23–S34. 10.1097/PHM.0000000000000647 28059876

[CIT0011] Hanass-Hancock, J., Nene, S., Deghaye, N. & Pillay, S., 2017, ‘“These are not luxuries, it is essential for access to life”: Disability-related out-of-pocket costs as a driver of economic vulnerability in South Africa’, *African Journal of Disability* 6, 280. 10.4102/ajod.v6i0.280 28730066 PMC5502471

[CIT0012] Joseph, C., Delcarme, A., Vlok, I., Wahman, K., Phillips, J. & Nilsson Wikmar, L., 2015, ‘Incidence and aetiology of traumatic spinal cord injury in Cape Town, South Africa: A prospective, population-based study’, *Spinal Cord* 53(9), 692–696. 10.1038/sc.2015.51 25823800

[CIT0013] Malvaso, A. & Kang, W., 2022, ‘The relationship between areas of life satisfaction, personality, and overall life satisfaction: An integrated account’, *Frontiers in Psychology* 13, 894610. 10.3389/fpsyg.2022.894610 36211891 PMC9532945

[CIT0014] Moller, P.H. & Taule, T., 2012, ‘Vocational rehabilitation services in South Africa: A survey of rehabilitation counsellors’, *Work* 42(1), 127–134.

[CIT0015] Naidoo, S., 2012, ‘The South African national health insurance: A revolution in health-care delivery!’, *Journal of Public Health* 34(1), 149–150. 10.1093/pubmed/fds008 22362968

[CIT0016] Nizeyimana, E., Louw, Q.A., Phillips, J. & Joseph, C., 2024, ‘Stakeholders’ perspectives on community reintegration after spinal cord injury in South Africa’, *Rehabilitation Advances in Developing Health Systems* 1(1), 1–9. 10.4102/radhs.v1i1.4

[CIT0017] Petrovič, F. & Maturkanič, P., 2022, ‘The urban-rural dichotomy of quality of life’, *Sustainability* 14(14), 8658. 10.3390/su14148658

[CIT0018] Phillips, J., Braaf, J. & Joseph, C., 2018, ‘Another piece to the epidemiological puzzle of traumatic spinal cord injury in Cape Town, South Africa: A population-based study’, *South African Medical Journal* 108(12), 1051–1054. 10.7196/SAMJ.2018.v108i12.13134 30606291

[CIT0019] Shim, D.Y. & Park, S.S., 2020, ‘A comparative study on life satisfaction on the disabled elderly and the disabled middle aged’, *Medico-Legal Update* 20(1), 2155–2160.

[CIT0020] Singh, J.A. & Cleveland, J.D., 2020, ‘Socioeconomic status and healthcare access are associated with healthcare utilization after knee arthroplasty: A US national cohort study’, *Joint Bone Spine* 87(2), 157–162. 10.1016/j.jbspin.2019.11.007 31811933

[CIT0021] Statistics South Africa, 2014, *General household survey 2014*, Statistics South Africa, Pretoria.

[CIT0022] Sullivan, G.M. & Artino Jr, A.R., 2013, ‘Analyzing and interpreting data from Likert-type scales’, *Journal of Graduate Medical Education* 5(4), 541–542. 10.4300/JGME-5-4-18 24454995 PMC3886444

[CIT0023] Theofilou, P., 2013, ‘Quality of life: Definition and measurement’, *Europe’s Journal of Psychology* 9(1), 150–162. 10.5964/ejop.v9i1.337.

[CIT0024] Vanderschuren, M.J. & Nnene, O.A., 2021, ‘Inclusive planning: African policy inventory and South African mobility case study on the exclusion of persons with disabilities’, *Health Research Policy and Systems* 19(1), 1–12. 10.1186/s12961-021-00775-1 34503537 PMC8428119

[CIT0025] Vergunst, R., Swartz, L., Hem, K.G., Eide, A.H., Mannan, H., MacLachlan, M. et al., 2017, ‘Access to health care for persons with disabilities in rural South Africa’, *BMC Health Services Research* 17, 1–8. 10.1186/s12913-017-2674-5 29149852 PMC5693516

[CIT0026] Vergunst, R., Swartz, L., Mji, G., MacLachlan, M. & Mannan, H., 2015, ‘“You must carry your wheelchair” – Barriers to accessing healthcare in a South African rural area’, *Global Health Action* 8(1), 29003. 10.3402/gha.v8.29003 26434691 PMC4592846

[CIT0027] Visagie, S., Eide, A.H., Dyrstad, K., Mannan, H., Swartz, L., Schneider, M. et al., 2017, ‘Factors related to environmental barriers experienced by persons with and without disabilities in diverse African settings’, *PLoS One* 12(10), e0186342. 10.1371/journal.pone.0186342 29023578 PMC5638520

[CIT0028] Wang, Y., Zhang, Z., Gong, W., Lv, Z., Qi, J. et al., 2025, ‘Analysis and validation of programmed cell death genes associated with spinal cord injury progression based on bioinformatics and machine learning’, *International Immunopharmacology* 149, 114220. 10.1016/j.intimp.2025.114220 39929099

[CIT0029] Whoqol Group, 1998, ‘Development of the World Health Organization WHOQOL-BREF quality of life assessment’, *Psychological Medicine* 28(3), 551–558. 10.1017/S0033291798006667 9626712

[CIT0030] Young, M., 2016, *Private vs. public healthcare in South Africa*, viewed 01 March 2025, from https://scholarworks.wmich.edu/honorstheses/2741

